# Supplementary material for: A pilot study combining noninvasive spinal neuromodulation and activity-based neurorehabilitation therapy in children with cerebral palsy
Source: Nat Commun. 2022 Oct 5;13:5660. doi: 10.1038/s41467-022-33208-w (PMC9535012; doi:10.1038/s41467-022-33208-w)
Supplement: Supplementary file 2 — Description of Additional Supplementary Files [file 41467_2022_33208_MOESM2_ESM.pdf]

Description of Additional Supplementary Files

Supplementary Video 1: Supplementary of a representative child undergoing ABNT with SCiP and changes over time.
