# Supplementary material for: A pilot study combining noninvasive spinal neuromodulation and activity-based neurorehabilitation therapy in children with cerebral palsy
Source: Nat Commun. 2022 Oct 5;13:5660. doi: 10.1038/s41467-022-33208-w (PMC9535012; doi:10.1038/s41467-022-33208-w)
Supplement: Supplementary file 4 — Reporting Summary [file 41467_2022_33208_MOESM4_ESM.pdf]

nature portfolio

Corresponding author(s): Parag Gad , PhD

Last updated by author(s): Jun 30, 2022

# Reporting Summary

Nature Portfolio wishes to improve the reproducibility of the work that we publish. This form provides structure for consistency and transparency in reporting. For further information on Nature Portfolio policies, see our Editorial Policies and the Editorial Policy Checklist.

## Statistics

For all statistical analyses, confirm that the following items are present in the figure legend, table legend, main text, or Methods section.

| n/a | Confirmed | |
|---|---|---|
| ☐ | ☒ | The exact sample size (*n*) for each experimental group/condition, given as a discrete number and unit of measurement |
| ☐ | ☒ | A statement on whether measurements were taken from distinct samples or whether the same sample was measured repeatedly |
| ☐ | ☒ | The statistical test(s) used AND whether they are one- or two-sided<br>*Only common tests should be described solely by name; describe more complex techniques in the Methods section.* |
| ☒ | ☐ | A description of all covariates tested |
| ☐ | ☒ | A description of any assumptions or corrections, such as tests of normality and adjustment for multiple comparisons |
| ☐ | ☒ | A full description of the statistical parameters including central tendency (e.g. means) or other basic estimates (e.g. regression coefficient) AND variation (e.g. standard deviation) or associated estimates of uncertainty (e.g. confidence intervals) |
| ☐ | ☒ | For null hypothesis testing, the test statistic (e.g. *F*, *t*, *r*) with confidence intervals, effect sizes, degrees of freedom and *P* value noted<br>*Give P values as exact values whenever suitable.* |
| ☒ | ☐ | For Bayesian analysis, information on the choice of priors and Markov chain Monte Carlo settings |
| ☒ | ☐ | For hierarchical and complex designs, identification of the appropriate level for tests and full reporting of outcomes |
| ☒ | ☐ | Estimates of effect sizes (e.g. Cohen's *d*, Pearson's *r*), indicating how they were calculated |

*Our web collection on statistics for biologists contains articles on many of the points above.*

## Software and code

Policy information about availability of computer code

| | |
|---|---|
| Data collection | No external data was used. No software was used for data collection |
| Data analysis | Graphpad Prism 7 was used to analyze data |

For manuscripts utilizing custom algorithms or software that are central to the research but not yet described in published literature, software must be made available to editors and reviewers. We strongly encourage code deposition in a community repository (e.g. GitHub). See the Nature Portfolio guidelines for submitting code & software for further information.

## Data

Policy information about availability of data

All manuscripts must include a data availability statement. This statement should provide the following information, where applicable:

- Accession codes, unique identifiers, or web links for publicly available datasets
- A description of any restrictions on data availability
- For clinical datasets or third party data, please ensure that the statement adheres to our policy

All source data are included with the resubmission in an excel file.

## Human research participants

Policy information about studies involving human research participants and Sex and Gender in Research.

| | |
|---|---|
| Reporting on sex and gender | Gender data was collected and reported in the manuscript in the respective tables. No gender based sub-analysis were done since the treatment or pathology is not gender dependent. |
| Population characteristics | Pediatrics aged between 2 to 18 and diagnosis of cerebral palsy GMFCS levels I to V. |
| Recruitment | Subjects were recruited from our database of potential participants. They were screened based on when they first registered in our database. Ones that met the criteria were invited to be part of the study. |
| Ethics oversight | Central IRB (Advarra) approved the study |

Note that full information on the approval of the study protocol must also be provided in the manuscript.

# Field-specific reporting

Please select the one below that is the best fit for your research. If you are not sure, read the appropriate sections before making your selection.

- [x] Life sciences
- [ ] Behavioural & social sciences
- [ ] Ecological, evolutionary & environmental sciences

For a reference copy of the document with all sections, see nature.com/documents/nr-reporting-summary-flat.pdf

# Life sciences study design

All studies must disclose on these points even when the disclosure is negative.

| | |
|---|---|
| Sample size | No Sample size calculations were done. This was a first in human study. Post hoc analysis were conducted to determine if the changes were statistically and clinically significant. |
| Data exclusions | No data were excluded |
| Replication | Patients of varying severities were included to ensure replication across a hetrogenous cohort. Each participant was only tested on one occasion at baseline and at final evaluation. |
| Randomization | Not randomized. This is a single arm pilot study. |
| Blinding | Single blind. Patients and parents were blinded to stimulation intensities. Since this is a first in human, single arm pilot study, blinding the investigators was not required. |

# Reporting for specific materials, systems and methods

We require information from authors about some types of materials, experimental systems and methods used in many studies. Here, indicate whether each material, system or method listed is relevant to your study. If you are not sure if a list item applies to your research, read the appropriate section before selecting a response.

## Materials & experimental systems

| n/a | Involved in the study | |
|---|---|---|
| x | | Antibodies |
| x | | Eukaryotic cell lines |
| x | | Palaeontology and archaeology |
| x | | Animals and other organisms |
| | x | Clinical data |
| x | | Dual use research of concern |

## Methods

| n/a | Involved in the study | |
|---|---|---|
| x | | ChIP-seq |
| x | | Flow cytometry |
| x | | MRI-based neuroimaging |

## Clinical data

Policy information about clinical studies

All manuscripts should comply with the ICMJE guidelines for publication of clinical research and a completed CONSORT checklist must be included with all submissions.

| | |
|---|---|
| Clinical trial registration | NCT04882592 |
| Study protocol | Included in the supplementary materials |
| Data collection | Data were collected at the site where therapy was delivered and occurred between May 2021 and Feb 2022. |
| Outcomes | GMFM88 is the gold standard for sensorimotor function in CP and was used as the primary outcome. GMFM88 is an instrument with 88 tasks that are administered by a qualified and trained therapist and each task is scored from zero to three. |
